# Influence of wikipedia and other web resources on acute and critical care decisions. a web-based survey

**DOI:** 10.1186/2197-425X-3-S1-A867

**Published:** 2015-10-01

**Authors:** B Rössler, H Holldack, K Schebesta

**Affiliations:** Department of Anesthesia, Critical Care and Pain M, Medical University of Vienna, Vienna, Austria; Sir Charles Gairdner Hospital, Department of Anaesthesia, Perth, Australia

## Introduction

Physicians use the Internet to gather medical information. However, little is known about the use and influence of Wikipedia, Google and other non-scientific web resources in acute and critical care medicine.

## Objectives

Therefore, we conducted an online survey among anaesthetists and critical care providers to address the use of non-scientific web resources and their influence on decisions made.

## Methods

After approval by the research ethics boards of the collaborating centres, 1,124 members of the ÖGARI (Austrian Society of Anaesthesiology, Resuscitation and Intensive Care) and 953 members of ANZCA (Australian and New Zealand College of Anaesthetists) were invited to participate in this anonymous online survey.

Demographic data as well as previous use of web-based resources were collected. The overall impact of online media on decisions made in an acute and critical care setting were assessed using 5 point Likert-like scales and multiple-choice questions, where applicable.

## Results

In total, 372 participants completed the survey, of whom 62% were consultants and 34% were in training. 54% were working in an academic setting and 95% had Internet access at their workplace. In order to get a fast overview about a medical problem, physicians would prefer Google (32%) over Wikipedia (19%) UpToDate (18%), or PubMed (17%). 39% would, at least sometimes, base their medical decisions on non peer-reviewed resources. Wikipedia is used often or sometimes by 77% of the interns, 74% of residents, and 65% of consultants to get a fast overview of a medical problem. Consulting Wikipedia or Google first in order to get more information about the pathophysiology, drug dosage or diagnostic options in a rare medical condition was the choice of 66%, 10% or 34%, respectively.

## Conclusions

Certified specialists and physicians in training utilise non peer-reviewed resources and those sites impact medical decision making in acute and critical care in Austria and Australia.
